# The Janus faces of thyroid carcinoma

**DOI:** 10.20945/2359-3997000000001

**Published:** 2018-01-01

**Affiliations:** 1 Universidade Federal de São Paulo Universidade Federal de São Paulo Escola Paulista de Medicina Laboratório de Endocrinologia Molecular e Translacional, Disciplina de Endocrinologia e Metabologia, Departamento de Medicina São Paulo SP Brasil Ambulatório de Doenças Tiroidianas; Laboratório de Endocrinologia Molecular e Translacional, Disciplina de Endocrinologia e Metabologia, Departamento de Medicina, Escola Paulista de Medicina, Universidade Federal de São Paulo (EPM-Unifesp), São Paulo, SP, Brasil

Thyroid cancer is the most common endocrine neoplasia, and its incidence has increased dramatically in several countries during the last three decades (1). This phenomenon has been attributed to overdiagnosis due to a combination of improvements in new imaging techniques, especially high-resolution ultrasound, and increased access of patients to health care systems (2). Irrespective of controversies regarding whether the growing number of thyroid cancer diagnoses is attributable to overdiagnosis alone (2) or reflects an actual increase in cases (3-5), the majority of tumor recently diagnosed is comprised of small papillary carcinomas (PTCs), (1.5-2.0 cm or less in size). Fortunately, more than 90% of them are curable with total thyroidectomy complemented or not with radioiodine (RAI). Although most PTCs are considered to be low risk, there are cases in which the tumor, even if small, exhibits aggressive behavior, and distant metastases can be present at diagnosis.

In this issue of the *Archives of Endocrinology and Metabolism* (AE&M), these two faces of thyroid cancer were addressed in two well-written papers on patients with PTC in two Latin American countries.

Domínguez and cols. (6), retrospectively reviewed the medical records of 209 patients with papillary thyroid microcarcinoma (PTMC) from 2009 to 2013, with a median follow-up of 4.4 years. Overall, 90% of these cases involved female patients; moreover, despite stratification into a low-risk group, 88% of patients received RAI, a treatment that was provided in accordance with older guidelines (7) and had no impact on tumor recurrence/persistence. As expected, classical PTC was the predominant histology (78%), tumors were frequently unilateral (76.1%), 17.9% of tumors had minimal extrathyroidal extension (ETE), 16.7% of cases involved lymph node metastasis, and there were no distant metastases. According to the American Thyroid Association (ATA) guidelines 2009 risk recurrence stratification (7), 70.8% of tumors were classified as low risk; this percentage increased to 78.5% when the ATA's 2015 risk stratification was used (8). In addition, persistence/recurrence was extremely uncommon: 1.5% of patients exhibited biochemical persistence/recurrence, and 5.5% of patients exhibited structural persistence/recurrence. Patients with persistence/recurrence tended to be younger (p = 0.08) and to present with multifocal tumors (p = 0.07), but only ETE (univariate analysis, p = 0.019) and lymph node involvement (multivariate analysis, p = 0.001) were significantly associated with persistence/recurrence. Comparisons of stratification using the 7^th^ and 8^th^ AJCC/TNM systems (9) indicated that when switching from the former system to the latter system, the percentage of patients classified as stage I increased from 89% to 95.2% and the percentage of patients classified as stage II decreased from 10% to 4.8%. Unfortunately, only 33% of patients with recurrent/persistent disease underwent preoperative neck ultrasound, which could explain why persistent disease was found during early follow-up.

In general, the data described in that paper resemble those that have previously been reported in the literature (10,11). These data also emphasize the fact that thyroid cancer, especially PTMC, has an excellent prognosis and that correct risk stratification is of great importance in patient management, with the objective of providing more cost-effective patient treatment and avoiding overtreatment. Moreover, these data could encourage local experts to adopt more conservative measures, such as “watchful waiting”, in the management of low-risk patients, a measure that has already been suggested by other groups around the world (12-14).

The other face of thyroid cancer refers to distinct outcomes for patients with distant metastases, which are likely to be found in the lungs and bones. Fortunately, this condition is uncommon, occurring in less than 10% of patients with differentiated thyroid carcinoma (DTC); however, such metastases are the main cause of thyroid cancer-related deaths (15).

Another article in this issue of AE&M describes a well-executed retrospective study by Califano and cols. (16), who assessed patients with DTC and bone metastases (BMs) followed at 10 referral endocrinology centers in Argentina to evaluate epidemiology, clinical presentation, treatments, and outcomes. Out of 3,810 patients with DTC, 52 patients (1.3%) had BMs, which were diagnosed less than 6 months after diagnosis of the primary tumor in 46.2% of these patients and later during follow-up in the remaining 53.8% of patients. In contrast to low-risk microcarcinomas, for DTC in this series, PTC accounted for 57.6% of cases, and the follicular variant of thyroid cancer was more frequent. As expected, the majority of patients were classified as having a high risk of recurrence (70.2%) according to the ATA's 2009 guidelines (7). With respect to the extent of disease, BMs were isolated in < 25% of cases, whereas locoregional or other metastatic sites, mostly in the lung (94.4%), were present in the remaining cases. In order of frequency, metastatic sites for BM included the spine, pelvis, ribs, limbs, skull, clavicle and sternum. BMs were frequently symptomatic (65.4%), and pain was the most frequent clinical presentation (73.5%). The treatment modalities were as described by others (15) and included RAI, bisphosphonates, external beam radiotherapy and other therapies (such as tyrosine kinase inhibitor, doxorubicin, thalidomide and radiofrequency ablation). More than 50% of patients died of DTC-associated causes, most of which were related to Hurthle cell variants and fracture at presentation. However, less than one-third of deaths were directly related to BMs; the remaining deaths were caused by other complications, which were mostly related to respiratory events. The authors found a relatively high frequency of negative RAI uptake at the metastatic site (42.3%); as described by Durante and cols. (15), patients who exhibited this characteristic more frequently died of DTC.

Califano and cols. also discussed the fact that when used as a solitary therapy, RAI therapy is rarely curative in patients with distant metastasis; only one patient achieved remission after this treatment. This statement is likely true for cases involving BMs. However, we recall that lung metastases were previously reported to be relatively responsive to RAI (15); thus, in a hypothetical condition in which RAI is associated with complete resection of isolated BM, this therapy is expected to improve overall survival. Certainly, as was well discussed by the authors, complete surgical resection can rarely be achieved if there are multiple BM sites. In such cases, palliative resection might be indicated more to improve quality of life than to impact survival. All of the patients in the examined series who were alive at the end of follow-up (36%) exhibited an incomplete structural response; this phenomenon is likely to be a consequence of broad bone involvement (isolated or combined) in this series. As stated by the authors, the correct management of patients with distant metastasis from DTC requires a multidisciplinary approach and currently remains a challenge; moreover, widespread research continues to be focused on searching for prognostic factors for cancer-specific mortality.

The aforementioned papers describe the two opposing faces of DTC. On one hand, the majority of patients with recently detected DTC have PTMC, which is associated with excellent prognosis and, in general, no mortality. For these diseases, appropriate risk stratification to determine which cancers require specific treatment and ongoing continuous restratification are essential to avoid overtreatment (17). For a specifically selected subgroup of low-risk patients, simple surveillance, with no specific treatment, has been proposed and stimulated (12-14). On the other hand, in rare cases involving metastatic DTC, this condition is frequently fatal or associated with persistent/recurrent disease, with great impact on the patient's quality of life and on healthcare systems worldwide. It has become increasingly challenging to treat this type of tumor. With respect to treatment, particularly for DTC refractory to RAI, molecular targeting therapies against most of the more common pathways involved in thyroid carcinogenesis have been proposed (18). Other targets include promoting immunological intervention, such as by increasing numbers of tumor-associated macrophages (TAMs) or by inhibiting factors associated with evading immunosurveillance (for example, in one study, an inhibitor of PD-1), two strategies that have shown promise (18).
